# A Comparative Study of the Efficacy of Intraoperative Intravenous Oxytocin and Intramuscular Oxytocin Versus Conventional Intramuscular Oxytocin for Third-Stage Labour in Elective Cesarean Section

**DOI:** 10.7759/cureus.35026

**Published:** 2023-02-15

**Authors:** Sasmita Behuria, Mahija Sahu, Minakshi Mohanty, Swayamprava Behera, Kirtirekha Mohapatra, Ranjita Patnaik, Satyajit Jena

**Affiliations:** 1 Obstetrics and Gynecology, Srirama Chandra Bhanj Medical College and Hospital, Cuttack, IND; 2 Obstetrics and Gynecology, Maharaja Krushna Chandra Gajapati Medical College and Hospital, Brahmapur, IND; 3 Community Medicine, Srirama Chandra Bhanj Medical College and Hospital, Cuttack, IND

**Keywords:** cesearian, labor, intravenous, intramascular, oxytocin

## Abstract

Objective

To study the efficacy of intraoperative IV oxytocin and intramuscular (IM) oxytocin versus conventional intramuscular oxytocin alone for active management of the third stage of labor in lower segment cesarean section (CS). The study was performed to determine the effect of 5 IU (International Unit) oxytocin infusion at the time of skin incision and that of 10 IU IM oxytocin infusion after delivery in reducing blood loss during and after CS, in comparison with the effect of administrating conventional 10 IU IM oxytocin in the same time period. In addition, it assessed the ability of the IV+IM oxytocin group to reduce the need for additional uterotonic as well as its safety determination and postoperative blood transfusion in CS.

Materials and methods

It is a randomized control study. The effect of 5 IU of oxytocin infusion at the time of skin incision and 10 IU of IM oxytocin (IV+IM) in reducing blood loss during and after the CS was compared to conventional 10 IU IM oxytocin.

Results

The study showed that the IV+IM group had a mean blood loss of 316.5 ± 74.36 ml, while the IM group had a mean loss of 403.90 ± 107.2 ml (p-value < 0.001) from placental delivery to the end of CS. A total of 90% of the patients in the IV+IM group had blood loss <50 ml compared to 95% of patients in the IM group who had a blood loss between 50 and 100 ml range from the end of cesarean to two hours postpartum. When total blood loss was compared in both groups, 84% of patients had a blood loss between 300 and 400 ml, compared to 81% of the patients in the IM group who had blood loss of 400-500 ml. Total blood loss in the IM group was 483.20 ± 115.86 ml, which was significantly higher compared to the IV group, 362.60 ± 78.07 ml (p-value=<0.001).

Conclusion

5IU oxytocin infusion at the time of skin incision and 10 IU IM oxytocin after delivery of the baby significantly reduced the amount of blood loss, need for blood transfusion, and additional uterotonics during and after lower segment CS.

## Introduction

Cesarean section (CS) is one of the most commonly performed obstetric procedures, and its rate continues to rise globally [1]. However, postpartum hemorrhage (PPH), as a possible effect of CS, is a significant cause of maternal morbidity and mortality worldwide. PPH accounts for nearly a quarter of all maternal deaths, more frequent with regard to lower segment Caesarean section (LSCS) (4%) than vaginal delivery (0.6%) [2-4]. Moreover, the related uterine atony, which complicates one in 20 deliveries, results in excessive blood loss. In addition, the standard physiological mechanism of the uterine contraction and retraction is disturbed in LSCS [2-4]. In this respect, the active management of the third stage of labor, including delayed cord clamping, controlled cord traction, and the administration of oxytocic drugs (such as ergometrine and oxytocin), has proved beneficial [5]. Therefore, the WHO recommends administering 10 IU (IM/IV) oxytocin to prevent PPH. Indeed, the prophylactic use of oxytocic agents has been shown to reduce the incidence of PPH by up to 40% [6]. Oxytocin is the most commonly used uterotonic agent in obstetrics because of its low cost and the rapid onset of its action. It is routinely administered during standard and operative deliveries to initiate and maintain adequate uterine contractions for minimizing blood loss [7-9]. Some studies indicate that oxytocin administration at the time of skin incision in elective LSCS reduces blood loss substantially [2-4]. Therefore, the present study was conducted to verify this hypothesis. It was performed to determine the effect of 5 IU oxytocin infusion at the time of skin incision and that of 10 IU IM oxytocin infusion after delivery in reducing blood loss during and after CS, compared with the effect of administrating conventional 10 IU IM oxytocin in the same time period. In addition, it assessed the ability of the IV+IM oxytocin group to reduce the need for additional uterotonic as well as its safety determination and postoperative blood transfusion in CS.

## Materials and methods

This randomized control study was performed in the Department of Obstetrics and Gynaecology, SCB Medical College and Hospital, Cuttack, Odisha, for a period of 12 months (from September 2020 to September 2021). Ethical approval for the study was taken from the institutional ethics committee (IEC) (ECR/84/inst/OR/2013/RR-20). For its investigation, 200 pregnant women who underwent LSCS before the onset of labor were included. The inclusion criteria for participant selection were women 18 years old or older, undergoing elective CS at term, admitted to the hospital before labor (at 37 weeks or more gestation), first-time pregnant, and having a singleton pregnancy. Those women who were admitted after the onset of labor or having fetal distress or abruptio placentae, as well as those with previous LSCS with doubtful scar integrity, prematurity, multiple pregnancies, heart disease with congestive cardiac failure, pulmonary edema, renal failure, Rh-negative pregnancy, diabetes mellitus and maternal jaundice with coagulopathy were excluded from this study. Informed consent was taken from all the participants before the commencement of the study. Detailed medical histories were taken, and clinical examinations were conducted for all the participants. After considering the inclusion and exclusion criteria, all eligible participants were alternately assigned either to the IV+IM group or the IM group. The following were the characteristics of both the groups: IV+IM group - subjects received 5 IU of oxytocin infusion in 500 ml normal saline (NS) at the time of skin incision and 10 IU of IM oxytocin after the delivery of the baby; IM group - subjects received only 10 IU of IM oxytocin after delivery. Further, the following were the requirements for measuring blood loss: standard gauze pads, a graduated measuring jar, and suction apparatus.

Clinical observations and laboratory examinations

Clinical Observations

Vital signs such as heart rate (HR), respiratory rate (RR), and blood pressure (BP) were checked preoperatively as well as after delivery. Blood loss was measured by the changes in weight and volume from the time of placental delivery to the hours after birth. Uterine contractility and placental separation were also observed. Moreover, the number of patients requiring additional uterotonic was noted. Neonatal manifestations were also assessed by the APGAR scores of the participants at one minute and five minutes. Significantly, all the above parameters were compared in both groups. Laboratory examinations, such as estimations of hemoglobin and hematocrit, before delivery and 24 hours after delivery were taken. All routine investigations were conducted if not done previously.

Quantitative Assessment of Blood Loss

Blood loss was assessed from the time of placental delivery till the end of CS as well as up to two hours postpartum, calculated by measuring the weights of all the soaked gauze pads, the operation theatre (OT) table sheets, and the amount of blood collected in the suction jar used during surgery. Notably, this study ignored the amount of blood loss that occurred prior to placental delivery, but total blood loss in both groups was taken into account.

Calculation of the Quantity of the Blood

The quantity of blood loss (ml) during surgery was calculated as the total weight of used and unused material (in grams or g) after surgery, minus the weight of all materials prior to surgery (g), plus the volume of blood collected in the suction container after placental delivery. The pads used were weighed after the completion of LSCS and two hours postpartum; the differences in their weights were also added to the calculation of blood loss. Further, assuming that 1 g equals 1 ml, the total blood loss was calculated.

Research Hypothesis

This study’s research hypothesis was that 5 IU of oxytocin infusion in NS 500 ml at the time of skin incision, along with 10 IU of IM oxytocin after delivery, decreases blood loss during and after LSCS, as compared to that incurred by the administration of only IM oxytocin. No major adverse effects were noted regarding IV+IM oxytocin compared to conventional IM oxytocin.

Statistical Analysis

Data for this study were collected and entered in Microsoft Excel 2007 and further analyzed in SPSS version 27 (IBM corporation). The associations between two categorical variables were assessed using either the Chi-squared test or Fisher’s exact test. The comparison of means between the two study groups was carried out by an independent sample t-test. The associations within the two groups were also determined using the paired sample t-test or the Wilcoxon signed-rank test. A p-value less than 0.05 was considered statistically significant.

## Results

The mean age group of participants in the IV+IM group was 24.49 ± 3.57 with a BMI of 24.49 ± 3.20 and gestational age of 39.31 ± 1.23, whereas the mean age group of participants in the IM group was 24.52 ± 3.51, with BMI of 24.58 ± 3.14 and gestational age of 39.29 ± 1.42. The mean age, BMI, and gestational age values were comparable in both groups (Table 1).

**Table 1:** Baseline characteristics of the study participants in both the group. IM: Intramuscular.

Baseline Characterstics	IV+IM group	IM group	𝝌^2 ^value/ t-value	P-value
Age (mean ± SD)	24.49 ± 3.57	24.52 ± 3.51	0.069	0.952
BMI (Mean ± SD)	24.49 ± 3.20	24.58 ± 3.14	0.069	0.838
Gestational age (Mean ± SD)	39.31 ± 1.23	39.29 ± 1.42	0.069	0.945

The blood loss from the time of placental delivery to the end of CS was significantly higher in the IM group (403.90 ± 107.2 ml) compared to the IV group (316.5 ± 74.36), with p-values <0.001. While only 53% of participants in the IV+IM group lost 300-500 ml of blood during this phase, 88% of those in the IM group lost 300-500 ml of blood during the same phase. This difference in proportion was also considered statistically significant (Table 2).

**Table 2:** Comparison of blood loss during placental delivery to end of Cesarian section (CS) between the group. IM: Intramuscular.

Blood loss in ml	IV+IM group N (%)	IM group N (%)	𝝌^2 ^value/ t-value	P-value
< 300 ml	45 (45.0)	4 (4.0)	46.59	<0.001
300-500 ml	53 (53.0)	88 (88.0)		
> 500 ml	2 (2.0)	8 (8.0)		
Mean ± SD	316.5 ± 74.36	403.90 ± 107.2	-6.699	<0.001

The mean blood loss from the end of CS to two hours postpartum was 46.10 ± 9.20 in the IV+IM group and 79.30 ± 17.71 ml in the IM group. Thus, the blood loss during postpartum was significantly higher in the IM group (p-value < 0.001) (Table 3).

**Table 3:** Comparison of blood loss during end of CS to two hours of postpartum period between the group. IM: Intramuscular; CS: Cesarean section.

Blood loss in ml	IV+IM group N (%)	IM group N (%)	𝝌^2 ^value/ t-value	P-value
< 50 ml	90 (90.0)	1 (1.0)	159.85	<0.001
50-100 ml	10 (10.0)	95 (95.0)		
> 100 ml	0 (0)	4 (4.0)		
Mean ± SD	46.10 ± 9.20	79.30 ± 17.71	-16.636	<0.001

Additionally, the total blood loss in the IM group was 483.20 ± 115.86 ml, significantly higher compared to that in the IV+IM group (362.60 ± 78.07 ml), with p-values <0.001. More than 80% of participants in the IV+IM group showed blood loss between 300 and 400 ml, while 81% of those in the IM group had blood loss between 400 and 500 ml. This difference in proportion was also considered statistically significant (Table 4).

**Table 4:** Comparison of total blood loss between the group. IM: Intramuscular.

Blood loss in ml	IV+IM group N (%)	IM group N (%)	𝝌^2 ^value/ t-value	P-value
< 300 ml	6 (6.0)	0 (0)	134.89	<0.001
300-400 ml	84 (84.0)	8 (8.0)		
400-500 ml	8 (8.0)	81 (81.0)		
> 500 ml	2 (2.0)	11 (11.0)		
Mean ± SD	362.60 ± 78.07	483.20 ± 115.86	-8.632	<0.001

The requirement for additional uterotonic was significantly higher in the IM group (75%) compared to the IV+IM group (25%). This difference in proportion was deemed statistically significant (p-value = 0.018) (Table 5).

**Table 5:** Comparison of additional uterotonic requirement in both the groups. IM: Intramuscular.

Additional uterotonic	IV+IM group N (%)	IM group N (%)	𝝌^2 ^value	P-value
Yes	5 (25.0)	15 (75.0)	5.556	0.018
No	95 (52.8)	85 (47.2)		

Out of all the participants requiring blood transfusion, 74.2% belonged to the IM group, whereas only 25.8% belonged to the IV+IM group. This difference in proportion was deemed statistically significant (p-value = 0.003). Also, no statistically significant difference was found regarding uterine artery ligation in both the study groups. Moreover, none of the participants had intraoperatively undergone a hysterectomy operation (Table 6).

**Table 6:** Comparison of blood transfusion, uterine artery ligation, hysterectomy in both the groups. IM: Intramuscular.

	IV+IM group N (%)	IM group N (%)	𝝌^2 ^value	P-value
Blood transfusion	8 (25.8)	23 (74.2)	8.589	0.003
Yes				
No	92 (54.4)	77 (45.6)		
Uterine artery ligation	1 (50.0)	1 (50.0)	0	1.00
Yes				
No	99 (50.0)	99 (50.0)		
Hysterectomy	-	-	-	-

The hemoglobin levels between the study groups were comparable before and after delivery (p-values > 0.05). Both the IV + IM and IM group participants showed decreased hemoglobin levels after delivery compared to those before delivery (p-values <0.05). Further, the comparison of hematocrit values before and after delivery showed significant differences between the study groups (p-values < 0.05) and also within the groups (p-values < 0.05) (Table 7).

**Table 7:** Comparison of laboratory parameters between the groups. IM: Intramuscular.

Laboratory parameters	IV+IM group (mean ± SD)	IM group (mean ± SD)	t-value	P-value
Hemoglobin				
Hemoglobin before delivery	10.70 ± 0.86	10.83 ± 0.89	-1.076	0.283
Hemoglobin after delivery	9.85 ± 2.73	9.66 ± 1.22	0.625	0.533
P-value within the group	0.002	<0.001		
Hematocrit				
Hematocrit before delivery	33.78 ± 2.86	32.91 ± 1.48	2.695	0.008
Hematocrit after delivery	32.38 ± 3.00	31.47 ± 1.47	2.732	0.007
P-value within the group	<0.001	<0.001		

Regarding the baby's weight, the respective APGAR scores at one minute and five minutes did not indicate any significant differences between the study groups (p-values > 0.05) (Table 8).

**Table 8:** Comparison of APGAR score and baby weight between the groups. IM: Intramuscular.

Duration in minutes	IV+IM group (mean ± SD)	IM group (mean ± SD)	t-value	P-value
Baby weight	2.90 ± 0.41	2.88 ± 0.38	0.414	0.679
APGAR at 1 min	8.81 ± 0.94	8.77 ± 0.90	0.306	0.760
APGAR at 5 mins	9.41 ± 0.75	9.39 ± 0.77	0.185	0.854

Notably, only one newborn in each group displayed respiratory distress and required NICU admission, with p-values >0.05 (Table 9).

**Table 9:** Comparison of NICU admission and respiratory distress between the group. IM: Intramuscular; NICU: Neonatal intensive care unit.

Variable	IV+IM group N (%)	IM group N (%)	𝝌^2 ^value	P-value
NICU admission	1 (50.0)	1 (50.0)	0	1.0
Respiratory distress	1 (50.0)	1 (50.0)	0	1.0

## Discussion

In the present study, both groups were comparable with regard to age, BMI, and gestational age. Keeping this in mind, the following related literature was used for the discussion. In a study similar to the present one, Takmaz T et al. used a different dose of oxytocin (20 IU/500 ml 0.9% saline). In turn, they found a significant reduction in blood loss during CS and the need for additional uterotonic in the group that started IV oxytocin infusion before fetal delivery [10]. Abdelaleem AA et al. also studied the effect of initiating oxytocin infusion before uterine incision during elective CS. They claimed that an early administration of oxytocin infusion would lead to a rapid onset of strong uterine contractions that would cause placental separation, thereby minimizing blood loss (432.7 ± 90.6 vs. 588.9 ± 96.3 ml respectively, p-values = 0.001) [11]. Moreover, Rajan VE et al. found that perioperative Syntocinon infusion of two units in NS 100 ml before uterine incision decreased intraoperative blood loss [12]. Furthermore, Tharwat AA et al. started a 15-minute IV drip with 10 IU/200 ml Ringer's lactate solution during the administration of anaesthesia, before skin incision, as compared to its use after the delivery of the fetus. As a result, the mean total blood loss was 340.3 ± 199.6 ml in group A and 484.3 ± 243.9 in group B (p-value < 0.001) [13]. In the present study, the IV+IM group showed significantly reduced bleeding from placental delivery to the end of CS. Also, the IV+IM and the IM groups reported mean blood loss values of 316.5 ± 74.36 ml and 403.90 ± 107.2 ml, respectively (p-value < 0.001). Plus, 88% of patients in the IM group reported blood loss between 300 and 500 ml, compared to 53% of patients in the IV + IM group claiming the same. Such a difference in proportion was considered statistically significant (p-value =<0.001). When the blood loss from the end of CS to two hours postpartum was compared between the study groups, 90% of the participants in the IV+IM group showed blood loss <50 ml, as compared to 95% of patients in the IM group who reported blood loss in the 50-100 ml range. The mean blood loss between the two groups was deemed statistically significant (p-values =<0.001). Further, when the total blood loss was compared in both the groups, 84% of participants in the IV+IM group reported blood loss between 300 and 400 ml, compared to 81% of the participants in the IM group who showed 400-500 ml blood loss. The total blood loss in the IM group was 483.20 ± 115.86 ml, significantly higher compared to that in the IV+IM group (362.60 ± 78.07 ml) (p-value =<0.001).

In this light, Aduloju OP et al. notably found that significantly more women required additional uterotonic after receiving oxytocin infusion following cord clamping than those receiving oxytocin infusion before uterine incision (p-value = 0.023) [14]. In this study, additional uterotonic was required by five patients in the IV+IM group as compared to 15 patients in the IM group. This difference was deemed statistically significant (p-values = 0.018). In addition, 74.2% of participants in the IM group needed blood transfusion compared to 25.8% in the IV+IM group. This difference was also considered statistically significant (p-value = 0.003). Plus, the mean hemoglobin levels between the two groups were comparable before and after delivery (p-values > 0.05). Within both the study groups, decreased hemoglobin levels were observed after delivery as compared to before delivery (p-values < 0.05). Moreover, the comparison of hematocrit levels before and after delivery between the two study groups showed a significant difference (p-values < 0.05), much like the hematocrit levels within the groups in the same time period (p-values < 0.05). Finally, regarding baby weight, the APGAR scores at one minute and five minutes showed no significant difference between the two study groups. Only one newborn child in each group had respiratory distress and required NICU admission (p-value > 0.05). In this light, Torloni MR et al. found that there was a significant reduction in blood loss and the need for additional uterotonic when oxytocin was given immediately before uterine incision compared to its administration after fetal delivery [15]. The limitation of this study is that this study was carried out in a single center, not multiple centers. Apart from IM and IV effects of oxytocin, bolus oxytocin should also be taken into account, and these comparisons should also be performed in participants with vaginal delivery to portray a better outcome regarding the efficiency of the delivery method of oxytocin.

## Conclusions

In summary, the IM+IV group participants reported significant reductions in the amount of blood loss, the need for additional uterotonic, and the necessity of blood transfusion during and after LSCS, as compared to the IM group. Crucially, fetal outcome, as evaluated by the APGAR scores, was not adversely affected by using 5 IU oxytocin infusion at the time of skin incision in LSCS. This finding can be used as a milestone intervention in preventing PPH, as evidenced in this study.
